# Incidence and risk factors of kinesiophobia in children following fracture surgery: a prospective cohort study

**DOI:** 10.3389/fped.2025.1536966

**Published:** 2025-11-24

**Authors:** Dan Xiao, Lanxing Li, Xin Lin, Haisu Li, Xiaoping Jiang

**Affiliations:** 1 Nursing Department, Children's Hospital of Chongqing Medical University, National Clinical Research Center for Child Health and Disorders, Ministry of Education Key Laboratory of Child Development and Disorders, Chongqing Key Laboratory of Pediatric Metabolism and Inflammatory Diseases, Chongqing, China; 2 Department of Orthopedics, Children's Hospital of Chongqing Medical University, Chongqing, China; 3 Department of Anesthesiology, Children's Hospital of Chongqing Medical University, Chongqing, China

**Keywords:** pain, kinesiophobia, children, fracture, personality traits, risk factors

## Abstract

**Objective:**

Kinesiophobia after fracture surgery in pediatric patients may negatively affect recovery. This study aims to investigate the incidence of kinesiophobia and identify associated risk factors in children following fracture surgery.

**Methods:**

This cohort study prospectively enrolled 176 pediatric fracture patients aged 7–15 years who received treatment at a tertiary A-level hospital between November 2023 and June 2024. Data collection included the General Information Questionnaire, Tampa Scale of Kinesiophobia (TSK-11), Pain Rating Scale, and Eysenck Personality Questionnaire (EPQ) to assess demographic characteristics, personality traits, family background, postoperative pain levels, and the incidence of kinesiophobia. Univariate screening through intergroup comparisons was performed, followed by logistic regression analysis to identify independent risk factors for kinesiophobia following fracture surgery in children.

**Results:**

The incidence of kinesiophobia among school-aged children post-fracture surgery was found to be 59.7% (105/176). Multivariate logistic regression analysis indicated that male gender [2.75 (1.23–6.15), OR (95% CI), *p* = 0.014], a history of prior fractures [6.62 (1.41–31.12), OR (95% CI), *p* = 0.017], moderate [4.82 (1.19–19.44), OR (95% CI), *p* = 0.027] and severe [5.14 (1.13–23.37), OR (95% CI), *p* = 0.034] postoperative pain, and a personality trait inclination towards neuroticism [1.12 (1.04–1.22), OR (95% CI), *p* = 0.004] were significant factors contributing to the development of kinesiophobia after surgery.

**Conclusion:**

The incidence of kinesiophobia is relatively high among school-aged children following fracture surgery. Healthcare providers should promptly identify cases of kinesiophobia and develop targeted care strategies based on identified risk factors to reduce its occurrence.

## 1 Introduction

Pediatric fractures are among the most common types of trauma, accounting for approximately 10%–25% of accidental injuries in children (1). The occurrence of fractures in children is closely linked to their active nature, developing motor skills, and environmental factors (2). In fracture treatment, the key principles include reduction, immobilization, and functional exercise, which are essential for restoring normal bone structure and function (3). Research has shown that timely and effective treatment can reduce recovery time and promote physical health in children (4). However, children often experience varying degrees of postoperative pain, leading them to resist essential rehabilitation exercises, which significantly decreases adherence to postoperative functional exercises (2, 5, 6). Poor adherence not only hinders the recovery of the affected limb but may also result in adverse outcomes such as restricted joint mobility, muscle atrophy, and prolonged recovery time, ultimately impacting the overall rehabilitation process and potentially affecting the child's mental health.

Asmundson et al. (7) proposed the Pediatric Fear-Avoidance Model of Chronic Pain, which explains this phenomenon of decreased compliance due to kinesiophobia. Kinesiophobia refers to an excessive and irrational fear of physical activity, triggered by heightened sensitivity to bodily symptoms or a lowered pain threshold following specific external stimuli or injuries (8, 9). This fear leads to intense anxiety when performing physical activities, and in some cases, patients may avoid daily activities due to fear of pain (10). As research advances, growing evidence suggests that kinesiophobia significantly impacts children's daily activities and social skills, potentially leading to chronic pain and even long-term functional disabilities, thereby seriously affecting their quality of life and mental health (11, 12). Yılmaz et al. (13) found a 64% incidence of kinesiophobia after bone tumor surgery in children, showing it is common in pediatric postoperative cases. Therefore, identifying and promptly addressing kinesiophobia is crucial for recovery after pediatric fracture surgery.

Although the impacts of kinesiophobia in children are well-recognized, its incidence and risk factors after pediatric fracture surgery remain unstudied. Therefore, this study employs a prospective cohort design to assess kinesiophobia using the Tampa Scale of Kinesiophobia (TSK-11), combined with comprehensive analysis of demographic characteristics, clinical indicators, and personality traits. The study aims to determine the incidence rate and identify key predictive factors of postoperative kinesiophobia in children with fractures, thereby providing a scientific basis for developing targeted intervention strategies for this patient population.

## 2 Materials and methods

### 2.1 Study subjects

This study was designed as a prospective cohort investigation that employed consecutive sampling to enroll pediatric patients (aged 7–15 years) who underwent fracture surgery and their primary caregivers at a tertiary-grade A children's hospital in Chongqing between November 2023 and June 2024.

### 2.2 Inclusion and exclusion criteria

Inclusion Criteria for Pediatric Patients: (1) children aged 7–15 years, based on the definition of a child provided by the International Convention on the Rights of the Child. (2) Patients clinically diagnosed with a fracture and admitted for surgical treatment. (3) Patients with basic reading and writing skills, capable of normal communication with the researcher. (4) Patients with informed consent from their guardians, and who voluntarily agreed to participate in the study.

Exclusion Criteria for Pediatric Patients: (1) patients in critical condition or requiring protective isolation. (2) Patients with mental or cognitive impairments. (3) Patients or their caregivers who were unwilling to participate in the study.

Inclusion Criteria for Caregivers: (1) caregivers aged 18 years or older. (2) Caregivers who provide more than 4 h of daily unpaid care to the child. (3) Caregivers with normal communication and comprehension abilities. (4) Caregivers who voluntarily agreed to participate and signed an informed consent form.

Exclusion Criteria for Caregivers: (1) caregivers with severe cardiopulmonary diseases, a history of psychiatric disorders, impaired consciousness, or intellectual disabilities, rendering them unable to complete study tasks.

The withdrawal and termination criteria included the request of the child or caregiver to discontinue participation. This study was approved by the Ethics Committee of our hospital (Approval No.: 2023-418) and registered in the Chinese Clinical Trial Registry (Registration Number: ChiCTR2400093049). All participants provided signed informed consent prior to participation, in compliance with the Declaration of Helsinki.

### 2.3 Sample size calculation

**Figure 1:**
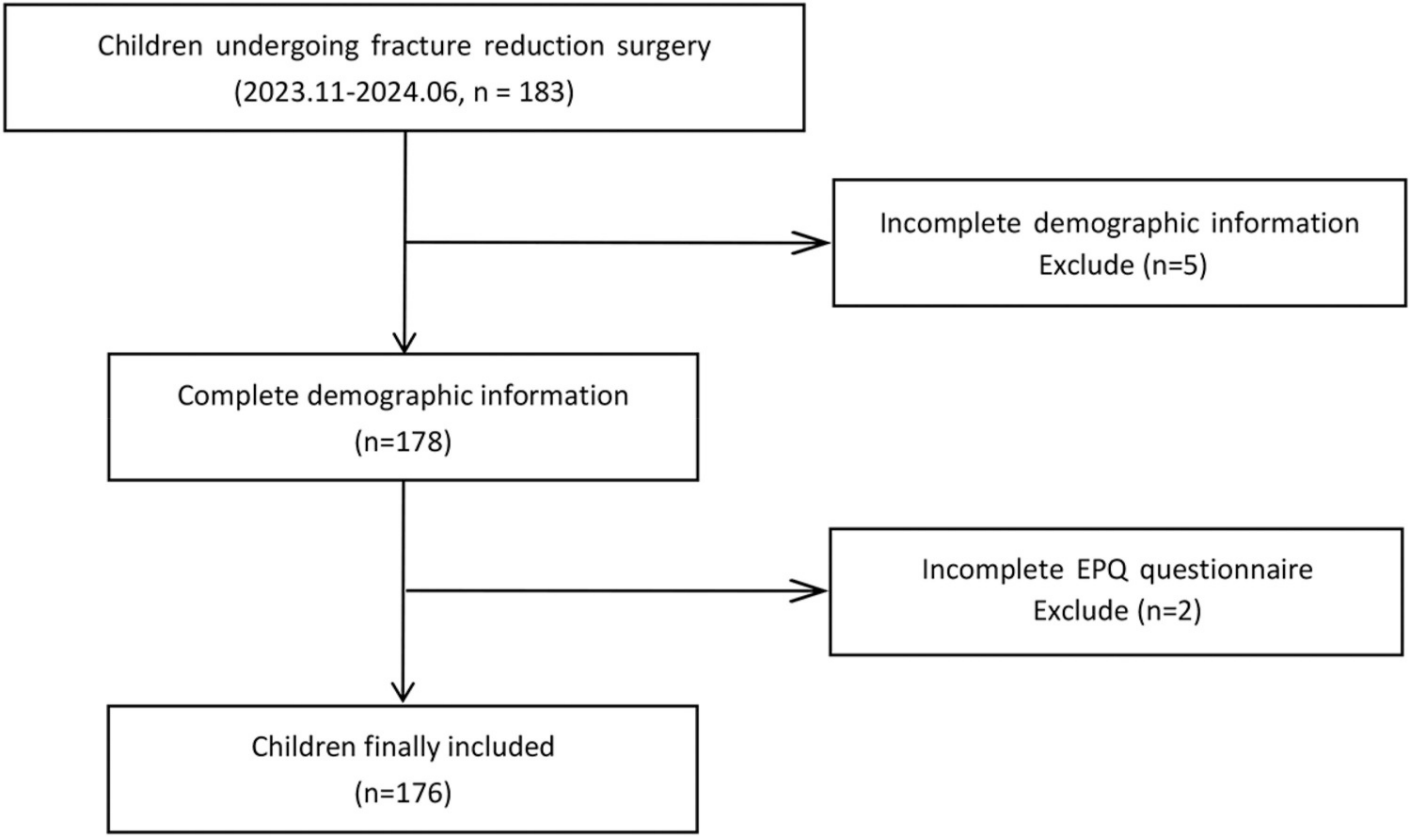
The study flow chart.

Based on Kendall's rough estimation method for sample size, the required sample size should be at least 5–10 times the number of variables (14). With a total of 20 variables in this study, the minimum sample size needed ranges from 100 to 200 participants. Considering a 10% rate of invalid questionnaires, the minimum sample size was adjusted to 110 participants. Ultimately, this study collected a total of 183 samples using consecutive sampling. After excluding 5 samples due to missing demographic information and 2 samples due to missing EPQ questionnaire data, 176 valid samples were included in the final analysis (see Figure 1).

### 2.4 Research tools

General Information Questionnaire: Based on a review of the literature and group discussions, the researchers designed and compiled a data collection form that includes factors potentially influencing postoperative pain and kinesiophobia in pediatric fracture patients. The questionnaire is divided into two main sections: demographic information and disease-related information. The demographic section gathers details about the child and the caregiver. For the child, information includes age, gender, BMI, history of previous fractures, history of previous surgeries, and chronic pain level. For the caregiver, information includes their relationship to the child, age, residence, education level, marital status, employment status, and whether there is a co-caregiver. The disease-related section includes anesthesia method, fracture site, postoperative pain management, and duration of surgery.

Numerical Rating Scale (NRS): The Numerical Rating Scale consists of a long ruler marked with 11 numbers ranging from 0 to 10, where 0 indicates no pain and 10 indicates severe pain (15). The higher the number, the greater the pain intensity, categorized as follows: 0 indicates no pain; 1–3 indicates mild pain; 4–7 indicates moderate pain; and 8–10 indicates severe pain.

Tampa Scale of Kinesiophobia (TSK-11): The TSK was revised by Woby et al. (16) in 2005 based on the “Fear-Avoidance Model,” reducing it to 11 items that utilize a 4-point Likert scale, where scores range from 1 to 4, corresponding to “strongly disagree” to “strongly agree.” The total score ranges from 11 to 44, with a score greater than 26 indicating the presence of kinesiophobia. The scale has been adapted into Chinese by Cai et al. (17), who tested its reliability in patients undergoing total knee arthroplasty, finding a Cronbach's *α* coefficient of 0.883 and a test-retest reliability of 0.798, demonstrating good validity and reliability.

Eysenck Personality Questionnaire for Children (EPQ): The revised Primary Eysenck Personality Questionnaire by Gong et al. (18) was used. This questionnaire consists of three dimensions and four subscales (extraversion-introversion scale, neuroticism scale, psychoticism scale, and validity scale), with a total of 88 items. Responses are in a binary format, with “yes” scoring 1 point and “no” scoring 0 points. Items 4, 9, 11, 16, 20, 30, 40, 49, 66, 67, 70, 73, 77, 80, 82, and 87 are reverse-scored.

### 2.5 Data collection

Face-to-face data collection was systematically conducted on postoperative day 1 by trained researchers. Using standardized administration protocols with both electronic and paper-based formats (Demographic Questionnaire, NRS, TSK-11, and EPQ), researchers first evaluated comprehension capacity in eligible child-parent dyads before obtaining informed consent for on-site questionnaire completion. Throughout administration, researchers maintained neutral clarification practices to prevent response bias. Caregiver-reported demographic/clinical information underwent rigorous cross-verification with institutional medical records. From initially distributed 183 questionnaires, meticulous quality control procedures identified and excluded 7 incomplete responses, yielding 176 analyzable datasets (96.2% validity rate). To preserve methodological integrity, all data collectors remained blinded to subsequent analysis phases.

### 2.6 Data analysis

Data from the questionnaires were exported to Excel and analyzed using SPSS 26.0 statistical software. Normally distributed continuous data with equal variances were expressed as mean ± standard deviation, while non-normally distributed data were presented as median and interquartile range. Categorical data were described using frequency and percentage. Differences between groups were analyzed using the chi-square test or Fisher's exact probability method. First, univariate screening was performed, and variables with statistical significance (*P* < 0.05) in the univariate analysis were included in the multivariate logistic regression model to explore the influencing factors of kinesiophobia in school-aged children with fractures. Variance inflation factor (VIF) values were calculated, with VIF < 5 indicating no significant multicollinearity among the variables. A *p*-value of less than 0.05 was considered statistically significant.

## 3 Results

### 3.1 Basic information of the patients

A total of 176 pediatric fracture patients were included in the study, among whom 105 developed kinesiophobia after surgery, resulting in an incidence rate of 59.7%. Significant differences were observed between the groups with and without kinesiophobia in terms of gender, history of prior fractures, TSK scores, and pain levels on the first postoperative day (*P* < 0.05). Male patients with a history of previous fractures and higher levels of postoperative pain were more likely to develop kinesiophobia. Detailed information regarding the patients' demographics is presented in Table 1.

**Table 1:** Demographic characteristics of pediatric patients.

Variables	Total (*n* = 176)	Group non-kinesiophobia (*n* = 71)	Group kinesiophobia (*n* = 105)	*P* value
Gender, *n* (%)				0.003
Male	132 (75.00)	45 (63.38)	87 (82.86)	
Female	44 (25.00)	26 (36.62)	18 (17.14)	
Age (years), M (Q1, Q3)	10.00 (8.00, 12.00)	10.00 (8.00, 12.00)	10.00 (8.00, 12.00)	0.972
BMI, *n* (%)				0.417
Underweight	17 (9.66)	5 (7.04)	12 (11.43)	
Normal weight	114 (64.77)	51 (71.83)	63 (60.00)	
Overweight	23 (13.07)	7 (9.86)	16 (15.24)	
Obesity	22 (12.50)	8 (11.27)	14 (13.33)	
History of previous fractures, *n* (%)				<.001
No	152 (86.36)	69 (97.18)	83 (79.05)	
Yes	24 (13.64)	2 (2.82)	22 (20.95)	
History of previous surgeries, *n* (%)				0.963
No	141 (80.11)	57 (80.28)	84 (80.00)	
Yes	35 (19.89)	14 (19.72)	21 (20.00)	
Chronic pain level, *n* (%)				0.500
Mild	6 (3.41)	1 (1.41)	5 (4.76)	
Moderate	168 (95.45)	69 (97.18)	99 (94.29)	
Severe	2 (1.14)	1 (1.41)	1 (0.95)	
Anesthesia method, *n* (%)				0.436
General + local anesthesia	170 (96.59)	70 (98.59)	100 (95.24)	
General anesthesia	6 (3.41)	1 (1.41)	5 (4.76)	
Fracture site, *n* (%)				0.280
Upper limb	126 (71.59)	54 (76.06)	72 (68.57)	
Lower limb	50 (28.41)	17 (23.94)	33 (31.43)	
Analgesia method, *n* (%)				0.636
Pain pump	171 (97.16)	68 (95.77)	103 (98.10)	
Oral pain medication	2 (1.14)	1 (1.41)	1 (0.95)	
None	3 (1.70)	2 (2.82)	1 (0.95)	
Surgery duration (min), M (Q_1_, Q_3_)	60.00 (45.00, 85.00)	60.00 (50.00, 82.00)	62.00 (43.00, 90.00)	0.940
Postoperative day 1 pain level, *n* (%)				0.010
Mild	15 (8.52)	11 (15.49)	4 (3.81)	
Moderate	113 (64.20)	46 (64.79)	67 (63.81)	
Severe	48 (27.27)	14 (19.72)	34 (32.38)	
TSK score, M (Q_1_, Q_3_)	28.00 (24.75, 30.00)	24.00 (22.00, 25.00)	29.00 (28.00, 31.00)	<.001

Data are shown as median (IQR) or Number (%).

TSK, Tampa scale for kinesiophobia.

### 3.2 Family background and personality traits of the patients

There were no statistically significant differences between the two groups regarding their family backgrounds. However, patients in the kinesiophobia group scored higher in the neuroticism and psychoticism dimensions of the Eysenck Personality Questionnaire (EPQ) (*P* < 0.05). Detailed information on the family background and personality traits of the patients is provided in Table 2.

**Table 2:** Family circumstances and personality traits of the pediatric patients.

Variables	Total (*n* = 176)	Group non-kinesiophobia (*n* = 71)	Group kinesiophobia (*n* = 105)	*P* value
Caregiver role, *n* (%)				0.819
Father	52 (29.55)	22 (30.99)	30 (28.57)	
Mother	114 (64.77)	44 (61.97)	70 (66.67)	
Grandparents	7 (3.98)	3 (4.23)	4 (3.81)	
Maternal grandparents	2 (1.14)	1 (1.41)	1 (0.95)	
Other	1 (0.57)	1 (1.41)	0 (0.00)	
Caregiver age (years), M (Q1, Q3)	33.00 (26.75, 40.25)	32.00 (27.00, 38.00)	34.00 (26.00, 42.00)	0.616
Household location, *n* (%)				0.120
Urban area	133 (75.57)	58 (81.69)	75 (71.43)	
Rural area	43 (24.43)	13 (18.31)	30 (28.57)	
Caregiver educational level, *n* (%)				0.122
Primary school and below	19 (10.80)	6 (8.45)	13 (12.38)	
Junior high school	47 (26.70)	14 (19.72)	33 (31.43)	
Vocational/High school	43 (24.43)	24 (33.80)	19 (18.10)	
Associate/Bachelor's degree	58 (32.95)	24 (33.80)	34 (32.38)	
Graduate degree and above	9 (5.11)	3 (4.23)	6 (5.71)	
Family marital status, *n* (%)				0.500
Married	165 (93.75)	65 (91.55)	100 (95.24)	
Divorced	11 (6.25)	6 (8.45)	5 (4.76)	
Caregiver employment status, *n* (%)				0.830
Full-time	104 (59.09)	45 (63.38)	59 (56.19)	
Part-time	8 (4.55)	2 (2.82)	6 (5.71)	
Unemployed	32 (18.18)	11 (15.49)	21 (20.00)	
Retired	8 (4.55)	3 (4.23)	5 (4.76)	
Other Employment Status	24 (13.64)	10 (14.08)	14 (13.33)	
More than one caregiver, *n* (%)				0.415
No	35 (19.89)	12 (16.90)	23 (21.90)	
Yes	141 (80.11)	59 (83.10)	82 (78.10)	
Eysenck Personality Questionnaire Scale				
Extraversion, M (Q_1_, Q_3_)	17.00 (15.00, 19.00)	17.00 (15.00, 18.00)	17.00 (15.00, 19.00)	0.445
Neuroticism, M (Q_1_, Q_3_)	6.00 (3.00, 11.00)	4.00 (2.00, 7.00)	9.00 (3.00, 13.00)	<.001
Psychoticism, M (Q_1_, Q_3_)	2.00 (1.00, 4.00)	1.00 (0.50, 3.00)	2.00 (1.00, 4.00)	0.004
Lie scale, M (Q_1_, Q_3_)	15.00 (11.00, 18.00)	16.00 (12.00, 18.00)	15.00 (11.00, 18.00)	0.184

Data are shown as median (IQR) or Number (%).

### 3.3 Risk factors for kinesiophobia

Using the presence of kinesiophobia as the dependent variable, univariate logistic regression analysis was performed with patient demographics, family background, and personality traits as independent variables. The analysis indicated that being male [2.79 (1.39–5.63), OR (95% CI), *p* = 0.004], having a history of previous fractures [9.14 (2.08–40.24), OR (95% CI), *p* = 0.003], experiencing moderate [4.01 (1.20–13.36), OR (95% CI), *p* = 0.024] or severe pain [6.68 (1.82–24.57), OR (95% CI), *p* = 0.004] on the first postoperative day, and scoring higher on the neuroticism [1.13 (1.06–1.20), OR (95% CI), *p* 0.001] and psychoticism [1.26 (1.09–1.47), OR (95% CI), *p* = 0.003] dimensions were associated with an increased risk of kinesiophobia.

Multivariate logistic regression analysis was conducted by including the significant variables identified from the univariate analysis. The results indicated that being male [2.75 (1.23–6.15), OR (95% CI), *p* = 0.014], having a history of previous fractures [6.62 (1.41–31.12), OR (95% CI), *p* = 0.017], experiencing moderate [4.82 (1.19–19.44), OR (95% CI), *p* = 0.027] or severe [5.14 (1.13–23.37), OR (95% CI), *p* = 0.034] pain on the first postoperative day, and having a neuroticism tendency [1.12 (1.04–1.22), OR (95% CI), *p* = 0.004] were identified as in dependent risk factors for the development of kinesiophobia. Details are provided in Table 3.

**Table 3:** Univariate and multivariate logistic regression analysis.

Variables	Univariate logistic regression analysis		Multivariate logistic regression analysis		VIF
	OR (95% CI)	*P* value	OR (95% CI)	*P* value	
Sex					1.080
Female	Ref.	Ref.	Ref.	Ref.	
Male	2.79 (1.39∼5.63)	0.004	2.75 (1.23∼6.15)	0.014	
History of previous fractures					1.173
No	Ref.	Ref.	Ref.	Ref.	
Yes	9.14 (2.08∼40.24)	0.003	6.62 (1.41∼31.12)	0.017	
Postoperative day 1 pain level					1.157
Mild	Ref.	Ref.	Ref.	Ref.	
Moderate	4.01 (1.20∼13.36)	0.024	4.82 (1.19∼19.44)	0.027	
Severe	6.68 (1.82∼24.57)	0.004	5.14 (1.13∼23.37)	0.034	
Eysenck Personality Questionnaire Scale					
Neuroticism	1.13 (1.06∼1.20)	<.001	1.12 (1.04∼1.22)	0.004	1.488
Psychoticism	1.26 (1.09∼1.47)	0.003	1.06 (0.89∼1.27)	0.511	1.521

OR, odds ratio; CI, confidence Interval; VIF, variance inflation factor.

## 4 Discussion

This prospective cohort study found that the incidence of postoperative kinesiophobia in school-aged children with fractures was 59.7%. Male gender, history of previous fractures, pain level on postoperative day 1, and neuroticism were identified as independent risk factors for kinesiophobia. Our findings indicate that postoperative kinesiophobia is a common but frequently overlooked psychological barrier in pediatric fracture patients. When children worry that physical activity may trigger pain or reinjury, they tend to avoid normal activities and rehabilitation exercises (7, 18). Kinesiophobia can negatively affect activities of daily living, quality of life, and rehabilitation outcomes (19). Therefore, investigating the incidence of postoperative kinesiophobia and analyzing its independent risk factors are crucial for early recognition and targeted intervention by clinicians.

Two previous studies on postoperative kinesiophobia in children with bone tumors reported incidence rates of 93% and 64% (13, 20). The differing rates may be related to variations in postoperative acute pain management strategies, yet the consistently high incidence warrants attention. In the present study, kinesiophobia was assessed on postoperative day 1 using the TSK-11, yielding an incidence of 59.7%, which is lower than previously reported. Although 96.6% of surgeries employed general anesthesia combined with nerve block, and 97.2% of patients received postoperative analgesia via patient-controlled analgesia (PCA), indicating a relatively comprehensive perioperative analgesic regimen, the incidence of postoperative kinesiophobia remained high. Beyond optimizing perioperative analgesic protocols, kinesiophobia may also be influenced by intrinsic characteristics of the child.

Wolfe et al. (21) reported that boys are more susceptible to fractures than girls [1.12 (1.08–1.16), OR (95% CI), *p* < 0.001], consistent with our findings. In our study, male patients were more likely to develop postoperative kinesiophobia. This may be attributed to their typically higher pre-injury activity levels and greater physical engagement. The abrupt transition from high activity to immobilization after fracture may facilitate the formation of avoidance responses toward physical activity (22). Furthermore, societal expectations often demand boys to exhibit bravery and resilience; when injury challenges this self-perception, it may reinforce kinesiophobia during rehabilitation (23, 24). Therefore, personalized pain education addressing the psychophysiological changes in boys after injury is warranted.

Another significant risk factor is a history of previous fractures. Our study demonstrated that children with prior fracture history were more prone to postoperative kinesiophobia, likely due to excessive concern about reinjury based on past trauma (25). These children exhibit heightened sensitivity to perceived risks of repeat injury, resulting in stronger avoidance behaviors during rehabilitation. Additionally, memories of prior pain may exacerbate postoperative fear of physical activity, further delaying recovery. A history of previous fractures significantly increases the risk of postoperative kinesiophobia through multiple psychological mechanisms and should be prioritized in clinical interventions.

Postoperative pain severity exerts a critical influence on the development of kinesiophobia (26). Our study revealed that children experiencing moderate-to-severe pain were more likely to develop kinesiophobia, highlighting the profound psychological impact of pain. Pain is not only a somatic sensation but also an emotional and psychological burden. Persistent pain may lead to conditioned avoidance responses to physical activity in children (7). Moreover, postoperative pain can impair sleep quality, reduce emotional stability, and intensify kinesiophobia (27). Therefore, clinicians should prioritize perioperative pain management and implement effective analgesic strategies. Yang et al. (28) investigated the 90% effective concentration (EC90) of ropivacaine for interscalene brachial plexus block in pediatric upper-extremity fracture surgery, providing reference for precise dosing in children. Compared with single-agent analgesia, multimodal postoperative analgesia offers superior analgesic efficacy while minimizing adverse effects (29). Non-pharmacological interventions, such as cognitive behavioral therapy (CBT) and acceptance and commitment therapy (ACT) focused on assessment and exposure, can assist children in adapting to postoperative pain, reducing discomfort and fear (30–33). Concurrently, personalized strategies including analgesic medications, standardized pain assessment frequency, and tailored analgesic protocols can mitigate the risk of kinesiophobia.

Children with different personality traits exhibit varying responses to pain. Children with neurotic personality tendencies are more susceptible to postoperative kinesiophobia. Neurotic individuals are typically more sensitive to pain and discomfort, often accompanied by higher levels of anxiety and tension, which may reinforce avoidance behaviors during rehabilitation (34). Their fear of future uncertainty may lead to resistance against rehabilitation training, ultimately impairing functional recovery outcomes. Therefore, rehabilitation planning should consider personality traits, and clinicians should collaborate with family members to provide psychological support to alleviate the child's fear of physical activity (35, 36).

Despite these valuable findings, our study has several limitations. First, as a single-center study, regional bias may limit the generalizability of the results to other regions or hospital types. Second, as a prospective cohort study relying on medical records and questionnaires, confounding factors were not controlled. The timing of postoperative data collection and the subjective nature of survey data may compromise the accuracy and consistency of the findings. Future studies should conduct multicenter longitudinal or qualitative investigations to comprehensively analyze the etiology, processes, and influencing factors of fear responses in children, providing robust evidence for developing targeted interventions.

In summary, this study systematically reveals the high incidence of postoperative kinesiophobia in school-aged children with fractures and its key risk factors, including male gender, history of previous fractures, moderate-to-severe postoperative pain, and neuroticism. These findings emphasize that kinesiophobia is a common yet overlooked psychological barrier that poses a potential threat to rehabilitation outcomes. Therefore, early identification and intervention of kinesiophobia are essential to maximize the recovery of children's physical and mental health.

## 5 Conclusion

This study identified a 59.7% incidence of kinesiophobia in school-aged children post-fracture, with key risk factors including male gender, history of previous fractures, postoperative day 1 pain level, and neuroticism. Boys and children with higher pain levels were at significantly greater risk. Effective management should prioritize targeted pain control and psychological support to mitigate this risk. Future research should explore how family and caregiver dynamics influence children's fear responses to optimize care strategies.

## Data Availability

The original contributions presented in the study are included in the article/Supplementary Material, further inquiries can be directed to the corresponding authors.
